# Spatial distribution of intangible cultural heritage resources in China and its influencing factors

**DOI:** 10.1038/s41598-024-55454-2

**Published:** 2024-02-29

**Authors:** Zhongwu Zhang, Zheng Cui, Tongsheng Fan, Shiyun Ruan, Juemei Wu

**Affiliations:** 1 School of Geographical Sciences, Shanxi Normal University, Taiyuan, 030000 China; 2 School of Natural Resources and Geomatics, Nanning Normal Uniwersity, Nanning, 530001 China; 3 School of Software and Microelectronics, Peking University, Beijing, 100871 China; 4 School of Geographical Sciences, Liaoning Normal University, Dalian, 116029 China

**Keywords:** Intangible cultural heritage resources, Kernel density analysis, Global trend analysis, Geodetector, Spatial distribution, Influencing factors, Ecology, Environmental sciences, Environmental social sciences

## Abstract

Exploring the spatial distribution of China’s intangible cultural heritage resources and its influencing factors is an important foundation for their protection and development and a key step toward the integration of culture and tourism. To analyse the geographical distribution patterns of China’s 3610 intangible cultural heritage resources and their influencing factors, we comprehensively applied methods such as spatial analysis and geodetectors. The main findings are as follows: (1) In terms of spatial distribution, China’s intangible cultural heritage resources are unevenly distributed, with an overall agglomeration-type distribution. The distribution in the north‒south direction is more significant, with more resources in the east than in the west and more resources in the south than in the north. (2) In terms of the spatial distribution of various types of intangible cultural heritage sites, North and East China have always been areas with a high kernel density. (3) In terms of spatial trends, there is a clear correlation between the distribution of intangible cultural heritage resources and the state of economic development and historical and cultural heritage, i.e., the more economically developed and culturally rich a region is, the more resources of intangible cultural heritage there are. (4) The causes of the distribution of China’s intangible cultural heritage resources are complicated, the influence of social factors is much greater than that of natural factors, and multidimensional interactions have a relatively significant impact. This study is conducive to the planning and protection of China’s intangible cultural heritage resources at the national and regional levels and provides a reference for the sustainable development of China’s intangible cultural heritage resources.

## Introduction

Intangible cultural heritage is an important resource in China that unites the Chinese nation with an awareness of diligence and wisdom and has extremely high historical and cultural value. This cultural treasure, passed on by word of mouth and continued from generation to generation, not only is a symbol of national identity but also plays an indispensable role in deepening national identity and enhancing cultural soft power^1–3^. However, in the process of protecting and developing intangible cultural heritage, problems such as the serious destruction of resources, insufficient government support in some areas, and fewer inheritors of intangible cultural heritage have emerged^4–6^. In this context, it is highly important to explore the spatial distribution of China’s intangible cultural heritage resources, rationally plan the protection and development of related cultural and tourism industries among regions, promote the inheritance and adaptive use of intangible cultural heritage resources, and advance the integration of culture and tourism^7,8^.

The factors influencing the spatial distribution of intangible cultural heritage resources are complex, and exploring the dominant factors leading to the spatial distribution of intangible cultural heritage resources among different regions has become a strategic issue for cutting-edge research^9^. As a valuable cultural treasure for all mankind, intangible cultural heritage merits a study of the factors influencing its spatial distribution in China, which assists the development of global cultural diversity. By deepening the description and further explaining the concept of intangible cultural heritage resources, Western European countries, especially France, expect to draw attention to the spiritual value of these resources^10^. In the United States, the role of intangible cultural heritage resources in urban development is becoming increasingly important because of the targeted development of cultural resources^11^. South American countries such as Chile have attempted to integrate the tourism and cultural industries by using intangible cultural heritage techniques as a center of agritourism with local characteristics^12^. This study provides lessons and experiences for other countries and regions to harmonize the relationship between economic development and the protection of cultural resources. The research in this paper assists the protection and transmission of intangible cultural heritage and promotes global cultural prosperity.

Overseas research on intangible cultural heritage has developed rapidly, with fundamental research focusing on concepts^13^, typology^14^ and the protection and development of intangible cultural heritage resources^15,16^. With the further development of tourism, foreign studies have begun to pay attention to the combination of intangible cultural heritage and tourism and the spatial distribution of intangible cultural heritage resources^17,18^. In broad-scale studies, scholars discuss the distribution and development of industries related to intangible cultural heritage resources at the watershed, provincial and municipal levels. Usually analyzing the impact of intangible cultural heritage-related industries on people’s lives within a single spatial environment^19^, these studies enrich the quality of the related cultural tourism products and strengthen the educational function of intangible cultural heritage by revealing its history and culture^20^. However, single-scale studies of intangible cultural heritage resources lack comparisons between different perspectives on the same influencing factors^21^. This paper contains a comprehensive study at three levels—national, subregional (with seven geographic locations), and provincial—to compensate for the limitations of a single-scale perspective and simultaneously considers the impact of intangible cultural heritage resources on the development of tourism and cultural industries.

Research on intangible cultural heritage has focused mainly on the high-quality development of intangible cultural heritage resources^22,23^, the integration of intangible cultural heritage and the tourism industry^24^, and the cultural creativity of intangible cultural heritage^25^. Li et al.^26^ focused on the traditional villages in the Yellow River Basin and emphasized the important role of the evidence-based development of intangible cultural heritage resources in realizing rural revitalization, substantial development of the tourism industry and the protection of traditional villages. Li et al.^27^ theoretically analyzed the high-quality development of intangible cultural heritage resources in the Yangtze River Basin, and argued developers should pay attention to the regional planning of the relevant industries to form a synergistic effect. A large number of studies have statistically and characteristically demonstrated the positive significance of the rational planning and development of industries related to intangible cultural heritage resources for the revitalization of the countryside and the development of cultural resources from multiple data sources, such as yearbook data, the output value of tourism, and the number of high-quality cultural industries in the countryside. This paper analyses the factors affecting the spatial distribution of intangible cultural heritage resources under different geographical conditions and puts forward reasonable suggestions for the integration of culture and tourism, the synergistic regional development of cultural industries and the high-quality development of rural intangible cultural heritage resources.

Research on the spatial characteristics of intangible cultural heritage objects has mostly used methods such as kernel density analysis, standardized ellipses, imbalance indices and geographic concentration indices^28–30^. Research on the distribution of intangible cultural heritage resources has only a limited variety of research methodologies, and this paper highlights the changing trend of the continuous distribution of spatial data over a wide range of regions by increasing area of analysis. In terms of influencing factors, scholars at home and abroad have concentrated on methods such as the spatial Durbin model, geographically weighted regression, spatial superposition of data and structural equation modeling^31–33^, which lack analyses of the multiple factors and interactions that lead to differences in the spatial agglomeration of intangible cultural heritage resources in different regions. This paper not only identifies the dominant factors affecting the spatial distribution of intangible cultural heritage resources in China using geographic probes but also explores the interactions among the influencing factors.

In view of this, this paper takes China’s 34 provincial administrative divisions and seven geographic subregions as a multiscalar perspective and employs composite multifactor interaction analysis methods and geo-probes to reveal the spatial distribution characteristics and dynamic mechanisms of intangible cultural heritage resources from two levels, namely, the overall and typological levels, as well as the spatial types and structures, from a variety of perspectives. The specific objectives of this study are as follows: (1) comprehensive and objective presentation and mapping of the spatial distribution of intangible cultural heritage resources by means of an imbalance index, trend surface analysis and kernel density analysis. (2) Spatial analysis highlights the clustering of intangible cultural heritage resources at three spatial scales—the national level, the seven geographic subregions, and the 34 provincial administrative divisions—to enrich the research results in this field. (3) The dominant factors affecting the clustering of intangible cultural heritage resources in each region are analyzed through geodetector model factor detection and interaction detection and differentiated recommendations are proposed.

## Data sources and methods

### Research framework

**Figure 1:**
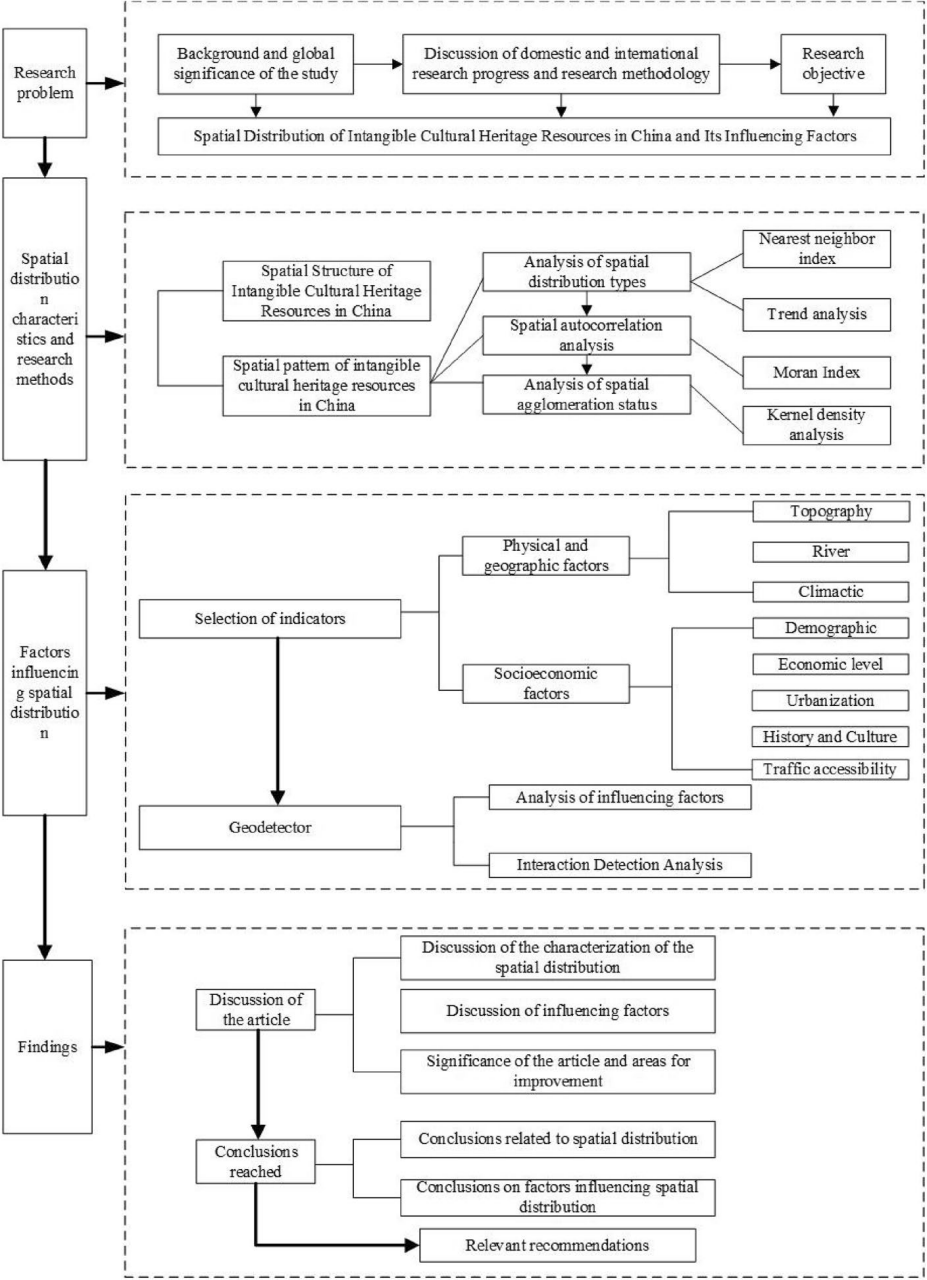
Research framework diagram.

A research framework for the spatial distribution of intangible cultural heritage resources and their influencing factors in China is proposed at different scales, including seven geographic subregions, both provincially and nationally (Fig. 1). Within this framework, the whole research process is divided into four main sections. The first section focuses on the description of the specific problem under study, its global significance and both its domestic and international progress (Section “Introduction”). Sections “Data sources and methods” and “Results and analysis” are the two broad themes of the study throughout the text, with Section “Data sources and methods” addressing the content and methodology of the study of spatial distribution (Section “Data Sources” and Section “Results and analysis”). The third section addresses the factors influencing the spatial distribution of intangible cultural heritage resources (Section “Influencing factors of spatial distribution”). The fourth section focuses on the discussion of the findings, conclusions and recommendations of this paper (Section “Discussion” and Section “Conclusions and recommendations”).

### Data sources

The data were obtained from the Intangible Cultural Heritage of China website (https://www.ihchina.cn). As of 2022, the Ministry of Culture and Tourism has published five batches of national intangible cultural heritage traditions totaling 3610 items, which will be used as the object of the study. The types of intangible cultural heritage will be classified with reference to the National Intangible Cultural Heritage List (https://www.ihchina.cn).

### Research methods

#### The nearest-point index

The closest neighbor index (R) is the proximity distance between points in a certain area and the identified element points. The distribution characteristics can be classified as random, uniform or agglomerative, and the size of the closest neighbor index can be used to analyze its spatial characteristics^31^.

1 $$ R = \overline{\gamma }_{a} /\overline{r}_{E} \quad \overline{{r_{E} }} = 1/2\sqrt {{n \mathord{\left/ {\vphantom {n A}} \right. \kern-0pt} A}} $$

where R is the nearest neighbor index, ${\overline{\gamma }}_{a}$ is the mean value of the actual proximity distance of each point, $\overline{{r }_{E}}$ denotes the theoretical proximity distance in a random distribution, n is the number of samples, A is the total range of the whole study area, R < 1, R = 1, and R > 1 denote the agglomerative distribution, the uniform distribution, and the random distribution, respectively, and the smaller the value of R is, the greater the degree of agglomeration.

#### Trend analysis

Trend surface analysis fits the values of intangible cultural heritage points with a function through global polynomials so that two-dimensional data can be expressed with three-dimensional curves to show the spatial trends distribution on a national scale^32^.

2 $${{\text{z}}}_{{\text{i}}}\left({{\text{x}}}_{{\text{i}}},{{\text{y}}}_{{\text{i}}}\right)={{\text{T}}}_{{\text{i}}}\left({{\text{x}}}_{{\text{i}}},{{\text{y}}}_{{\text{i}}}\right)+{{\text{h}}}_{{\text{i}}}$$

where $\left({x}_{i},{y}_{i}\right)$ is a coordinate point, ${z}_{i}\left({x}_{i},{y}_{i}\right)$ is the actual observed value of the ith intangible cultural heritage point, ${T}_{i}\left({x}_{i},{y}_{i}\right)$ is the fitted value of the trend surface, and ${h}_{i}$ is the deviation between the actual observed value and the fitted value.

#### Kernel density analysis

Kernel density analysis visualizes the degree of concentration of intangible cultural heritage points by calculating the density of points around the output raster^33,34^.

3 $${f}_{\left(x\right)}=\frac{1}{nh}\sum_{i=1}^{n}k\left(\frac{x-{x}_{i}}{h}\right)$$

where h is the bandwidth, n is the total number of intangible cultural heritage sites, $\left(x-{x}_{i}\right)$ denotes the distance from intangible cultural heritage site x to ${x}_{i}$, and k is a coefficient.

#### Spatial autocorrelation

Spatial autocorrelation effectively reveals whether there is an obvious correlation between the number of intangible cultural heritage sites in a certain region and the number of intangible cultural heritage sites in neighboring regions and tests whether there is a significant clustering characteristic of intangible cultural heritage sites.

Global Moran’s I index: indicates the extent to which areas with spatial proximity affect their neighboring areas.

4 $${\text{I}}=\frac{n\sum_{i=1}^{n}\sum_{j=1}^{n}{w}_{ij}\left({x}_{i}-\overline{x }\right)\left({x}_{j}-\overline{x }\right)}{\sum_{i=1}^{n}\sum_{j=1}^{n}{w}_{ij}\sum_{i=1}^{n}{\left({x}_{{\text{i}}}-\overline{x }\right)}^{2}}$$

where n is the number of observations in the region, ${w}_{ij}$ denotes the weight matrix of each intangible cultural heritage site i and intangible cultural heritage site $j$ in the study area, ${x}_{i}$ and ${x}_{j}$ are the values of the variable $x$ at positions i and $j$, respectively, and $\overline{x }$ is the mean value of the variable.

#### Geographical detector

The characteristics of two variables in a geodetector in a spatial structure are used to determine the links that exist between them^35^.

5 $${q=P}_{X,Y}=1-\frac{1}{n{\sigma }^{2}}\sum_{i=1}^{m}{n}_{i}{\sigma }_{i}^{2}$$

where Y is the number of intangible cultural heritage points, ${P}_{X,Y}$ is the influence of element X on Y, n is the number of observations, ${\sigma }^{2}$ is the variance across all regions, m is the number of strata in Y, and ${\sigma }_{i}^{2}$ is the subregional variance.

### Selection of indicators

According to the two perspectives of nature and humanity in expert opinions, to explore the influencing factors of intangible cultural heritage resources^36–39^, the two basic dimensions of the natural geographic environment and socioeconomics were selected. Nine indicators, such as topography, rivers, average temperature, population, and traffic, were extracted as the influencing factors (Table 1).

**Table 1:** Indicator construction of influencing factors of China’s intangible cultural heritage resources.

Dimension	Impact factor	Evaluation indicator	Data source
Physical geography	Topography X_1_	Average elevation (m)	Geospatial data cloud platform access
	River X_2_	River length (km)	China basic geographic information system data
	Climactic X_3_, X_4_	Annual rainfall (mm)	China national data network
		Average annual temperature ℃	
Socioeconomic Factors	Demographic X_5_	Number of people in the area (ten thousand people)	
	Economic level X_6_	Gross regional product (billions of Yuan)	
	Urbanization X_7_	Urban population as a proportion of total regional population	
	History and Culture X_8_	Based on a combination of indicators such as number of local museums, number of art groups, etc	Data from the national statistical yearbook of China
	Traffic accessibility X_9_	Road density	

## Results and analysis

### Spatial pattern of intangible cultural heritage resources in China

**Figure 2:**
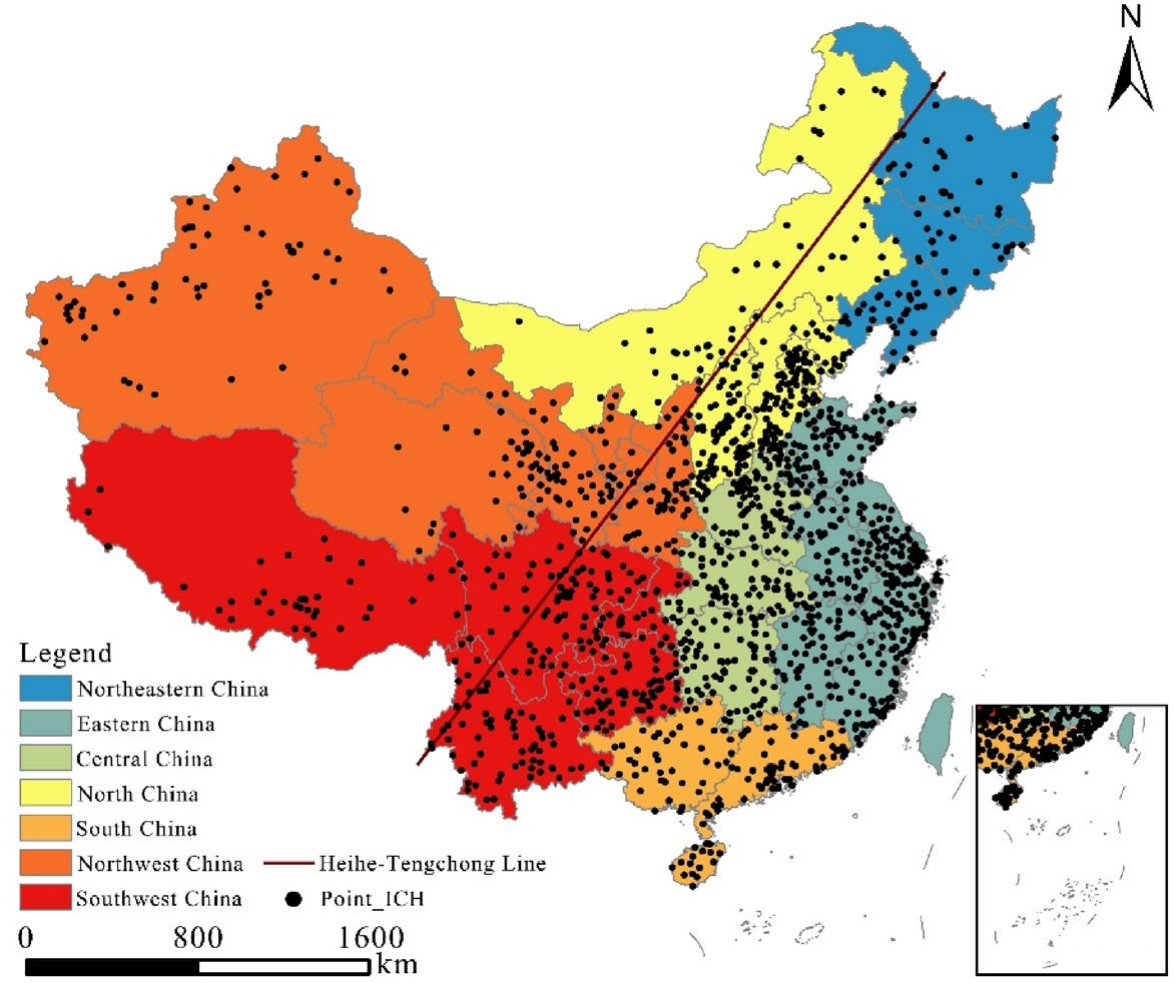
Spatial distribution of intangible cultural heritage resources in China. *Note* The map is based on the standard map No. GS (2020) 4631 downloaded from the standard map service website of the Bureau of Surveying, Mapping and Geographic Information of China and made through ArcGIS10.8, without modification of the bottom map boundaries. (http://bzdt.ch.mnr.gov.cn/) below Figs. 4, 5 and 6 production method is the same.

The spatial distribution of intangible cultural heritage resources in China generally shows a distribution of more resources in the east than the west (Fig. 2); in terms of geographical differences, the number of intangible cultural heritage resources is greater in the east and south, and the resources of intangible cultural heritage are also denser in these areas, using the Heihe-Tengchong line as a boundary. The population and urban distribution characteristics of the densely populated eastern and sparsely populated western regions have largely influenced the spatial distribution pattern of China’s intangible cultural heritage resources; these traditions are more common in the east than in the west and more common in the south than in the north.

At the scale of the seven geographic subregions, resources are heavily concentrated in southern, eastern and northern China. There are fewer resources in the northwest and northeast regions and more evenly distributed resources in central and southwest China.

### Spatial structure of intangible cultural heritage resources in China

#### Analysis of spatial distribution types

According to Eq. (1), the nearest-neighbor index (Table 2) is derived, with an R value of 0.24. An R value less than 1 indicates that the spatial distribution of intangible cultural heritage resources in China is clustered. The closest-neighbor ratios (R) of all types of intangible cultural heritage sites were lower than 0.6, so the spatial distribution of all types of intangible cultural heritage resources showed a clear clustering pattern (Table 2).

**Table 2:** Summary of average nearest neighbors of intangible cultural heritage.

Typology	Techniques	Fine art	Sports, amusement and acrobatics	Dance	Drama	Medicine	Music	Folk literature	Folkways	Dramatic balladry	Total
Quantity	629	417	166	356	473	182	431	251	492	213	3610
R-value	0.35	0.44	0.53	0.56	0.47	0.41	0.51	0.56	0.47	0.49	0.24
Z value	− 31.32	− 21.99	− 11.64	− 15.88	− 22.24	− 15.34	− 19.28	− 13.37	− 22.44	− 14.22	− 87.80
*P* value	0.00	0.00	0.00	0.00	0.00	0.00	0.00	0.00	0.00	0.00	0.00

**Figure 3:**
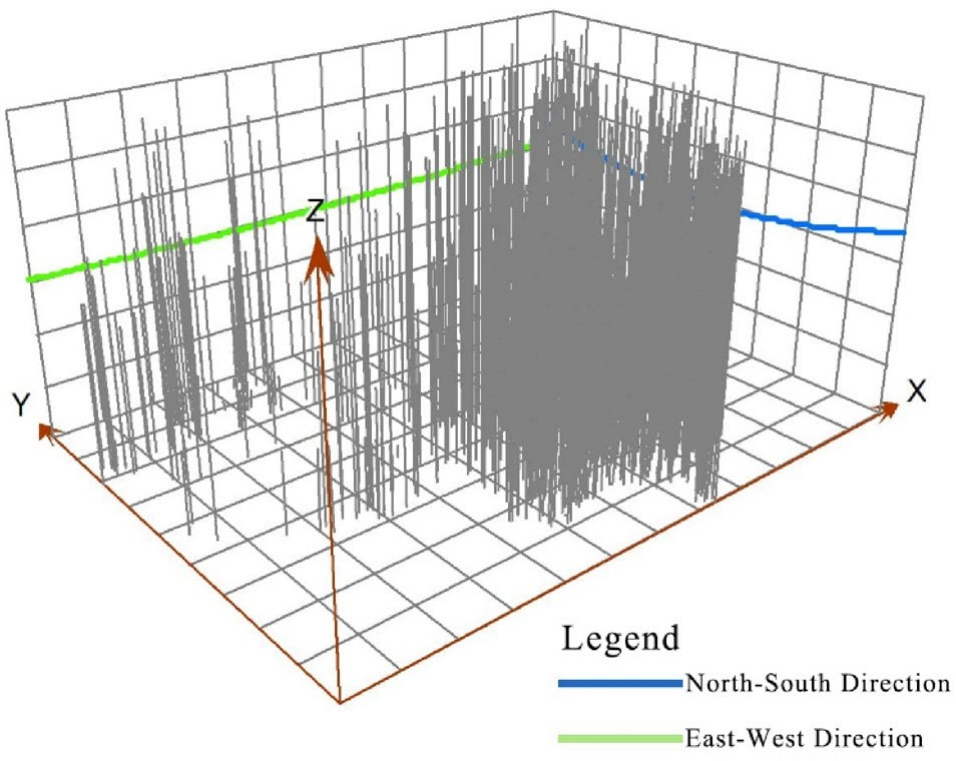
Surface fitting of general spatial trends in intangible cultural heritage resources.

Fitting the trend surface of China’s intangible cultural heritage resources according to Eq. (2), the spatial trend line basically maintains a pattern of more resourcesin the east than the west and more in the south than the north. The trend surface is steeper in the north‒south direction (Fig. 3), indicating that there is a more prominent north‒south distribution. China’s densely populated and economically developed regions are located in the southern and eastern regions of the country, so the distribution of China’s intangible cultural heritage resources is clearly correlated with the state of economic development. The more economically developed and culturally rich an area is, the more intangible cultural heritage resources there are.

#### Spatial autocorrelation analysis

The spatial Moran’s I index value of China’s intangible cultural heritage resources is 0.11, and the *p* value is 0.00, which passes the 1% significance test. This indicates that China’s intangible cultural heritage resources are clustered according to global autocorrelation, revealing a significant positive spatial correlation (Table 3). In other words, regions with high numbers of intangible cultural heritage resources are neighboring regions, as are regions with low numbers of intangible cultural heritage resources.

**Table 3:** Global spatial autocorrelation data table of China’s intangible cultural heritage resources.

Moran’s I	Z value	*P* value	Global distribution	Global autocorrelation
0.11	8.79	0.00	Clustering trends	Significant spatial positive correlation

#### Analysis of spatial agglomeration

**Figure 4:**
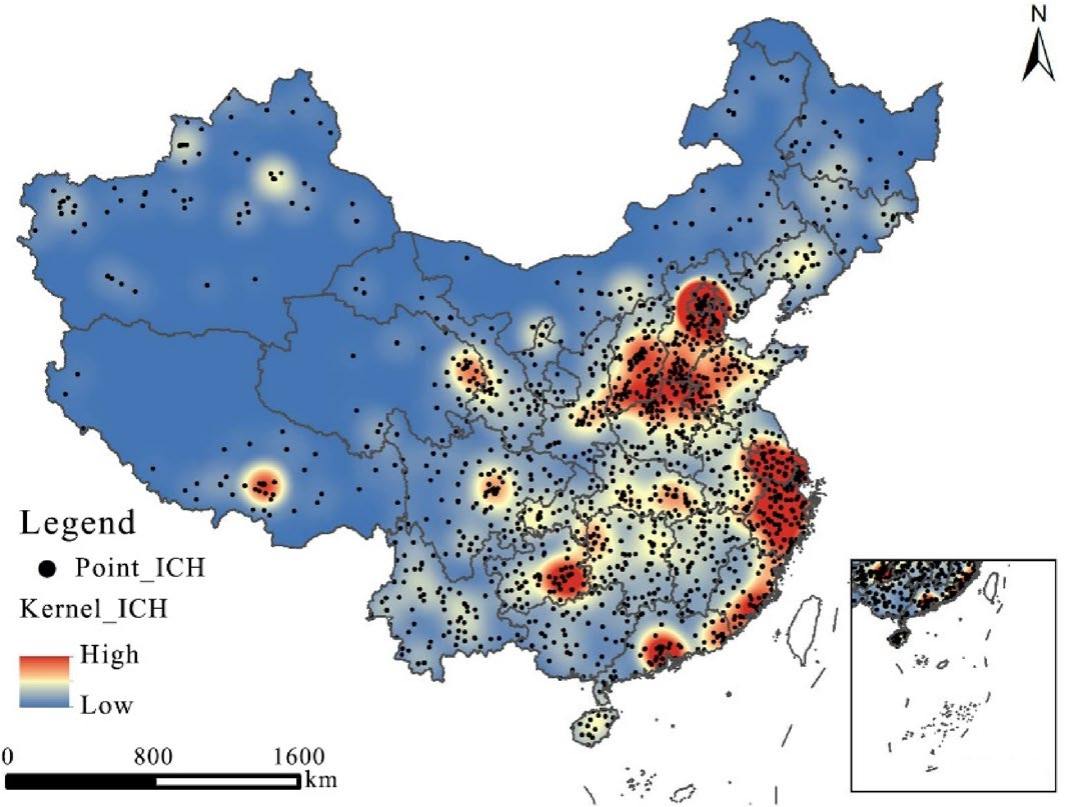
Overall distribution of kernel density of China’s intangible cultural heritage resources.

The distribution of China’s intangible cultural heritage resources has obviously agglomerated (Fig. 4), with a trend that can be characterized as “one group, one belt and many points”. In the formation of 2 high-density zones, North China centered on Beijing, Shanxi and Hebei and East China centered on Shanghai, Zhejiang and Jiangsu. In the formation of 2 subkernel density zones, South China centered on Guangzhou and Southeast China centered on Guizhou. There are also density zones around Lhasa and Chengdu.

The overall spatial distribution of intangible cultural heritage resources in China is uneven, and the characteristics of the distribution of resources are clearly related to rivers, economic development and historical and cultural heritage; i.e., the more economically developed and culturally rich a region is, the more intangible cultural heritage resources there are. At the scale of the seven geographic subregions, the 2 high- density zones form two regional clusters of intangible cultural heritage resources in North and East China. However, at the provincial and regional levels, previous authors have analyzed only the main factors influencing the spatial distribution of intangible cultural heritage resources across the whole region under study and have easily overlooked the dominant factors in the clustering of intangible cultural heritage resources in small-scale regions. For example, the intangible cultural heritage resource cluster in Tibet, centered on Lhasa, is formed predominantly by ethnicity; separately, the intangible cultural heritage resource cluster in Sichuan is predominantly formed by topography. These clusters of intangible cultural heritage resources at the provincial scale and the sporadic distribution of intangible cultural heritage resources in various regions should not be neglected.

**Figure 5:**
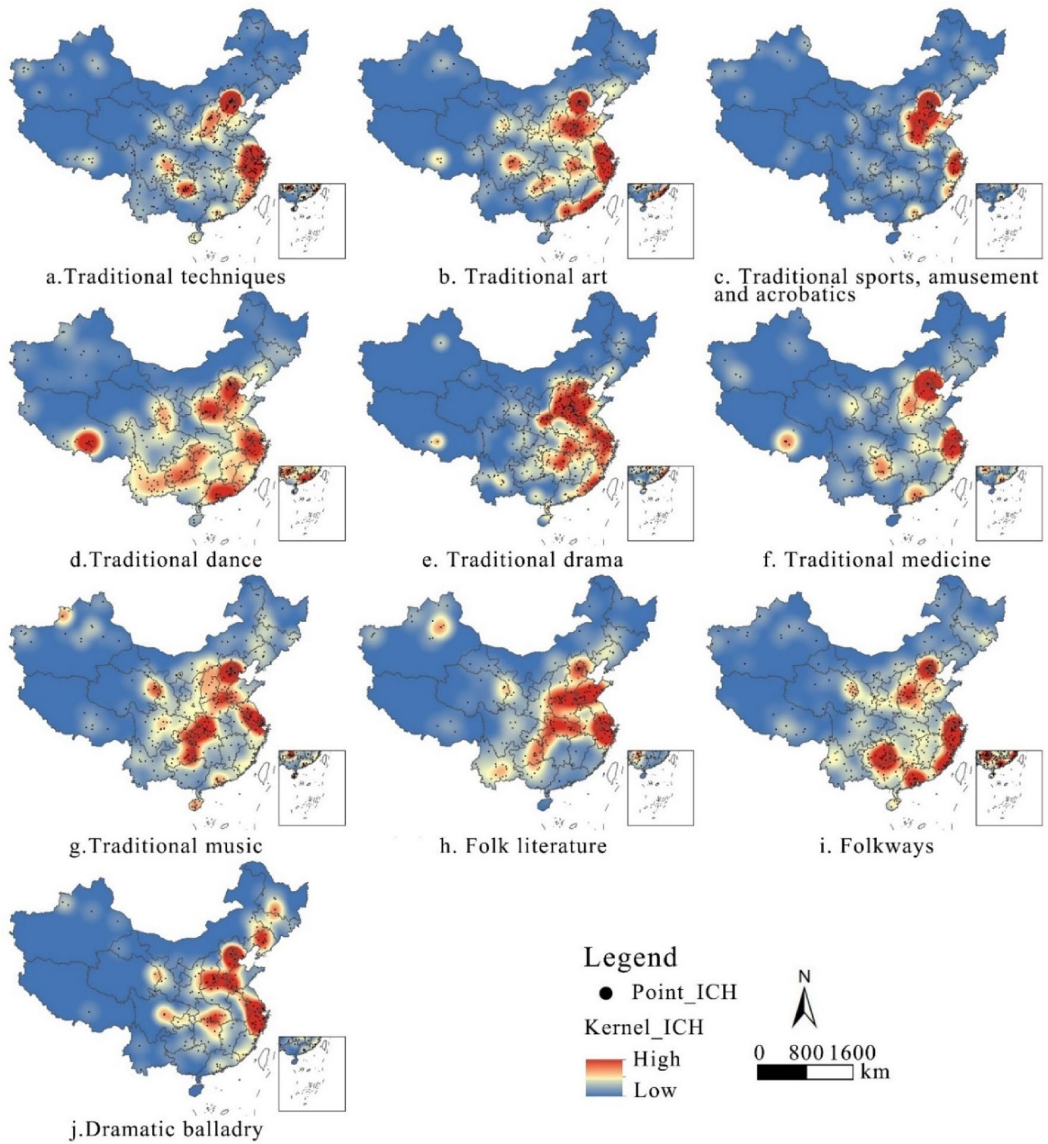
Distribution of kernel density for each type of China’s intangible cultural heritage resources.

Different types of intangible cultural heritage resources have different spatial clustering characteristics (Fig. 5), but the overall spatial pattern is still greater in the east than in the west. North and East China, centered on Beijing, Hebei and Shanxi, have always been areas of high kernel density. There are many types of intangible cultural heritage resources forming a dense cluster in Qiandongnan. Traditional dances form a dense cluster in the Tibetan region centered on Lhasa, and the genre is characterized by a banded distribution in the southwestern part of the country. Traditional skills, arts and music are densely clustered in the Sichuan region, centered on Chengdu. Traditional sports, games, acrobatics, and traditional theatre are mostly found in the northern part of the country, while traditional skills and folklore are mostly found in the southern part of the country.

## Influencing factors of spatial distribution

### Analysis of influencing factors

The explanatory variables at the provincial scale were imported into the geodetector model, and the R-language program was used to select the optimal discrete classification to derive the q value of the influence of each indicator on the spatial distribution of intangible cultural heritage resources in China. (Table 4).

**Table 4:** Explanatory power of influencing factors of China’s intangible cultural heritage resources.

Dimension	Physical and geographic factors				socioeconomic factors				
Targets	X1	X2	X3	X4	X5	X6	X7	X8	X9
Q Value	0.108**	0.109*	0.103**	0.206**	0.431**	0.519**	0.192*	0.486**	0.363**

*indicates significance at the 10% confidence level.

**indicates significance at the 1% confidence level.

The order of influence of factor detection was level of economic development > history and culture > population > accessibility > average annual temperature > urbanization > rivers > topography > annual precipitation. The average temperature and annual precipitation are part of the climatic conditions; therefore, these two indicators are considered together as climatic factors in the analysis. In terms of explanatory power, the degree of influence of socioeconomic factors on the spatial distribution of intangible cultural heritage resources in China is much greater than that of physical geography (Table 4).

**Figure 6:**
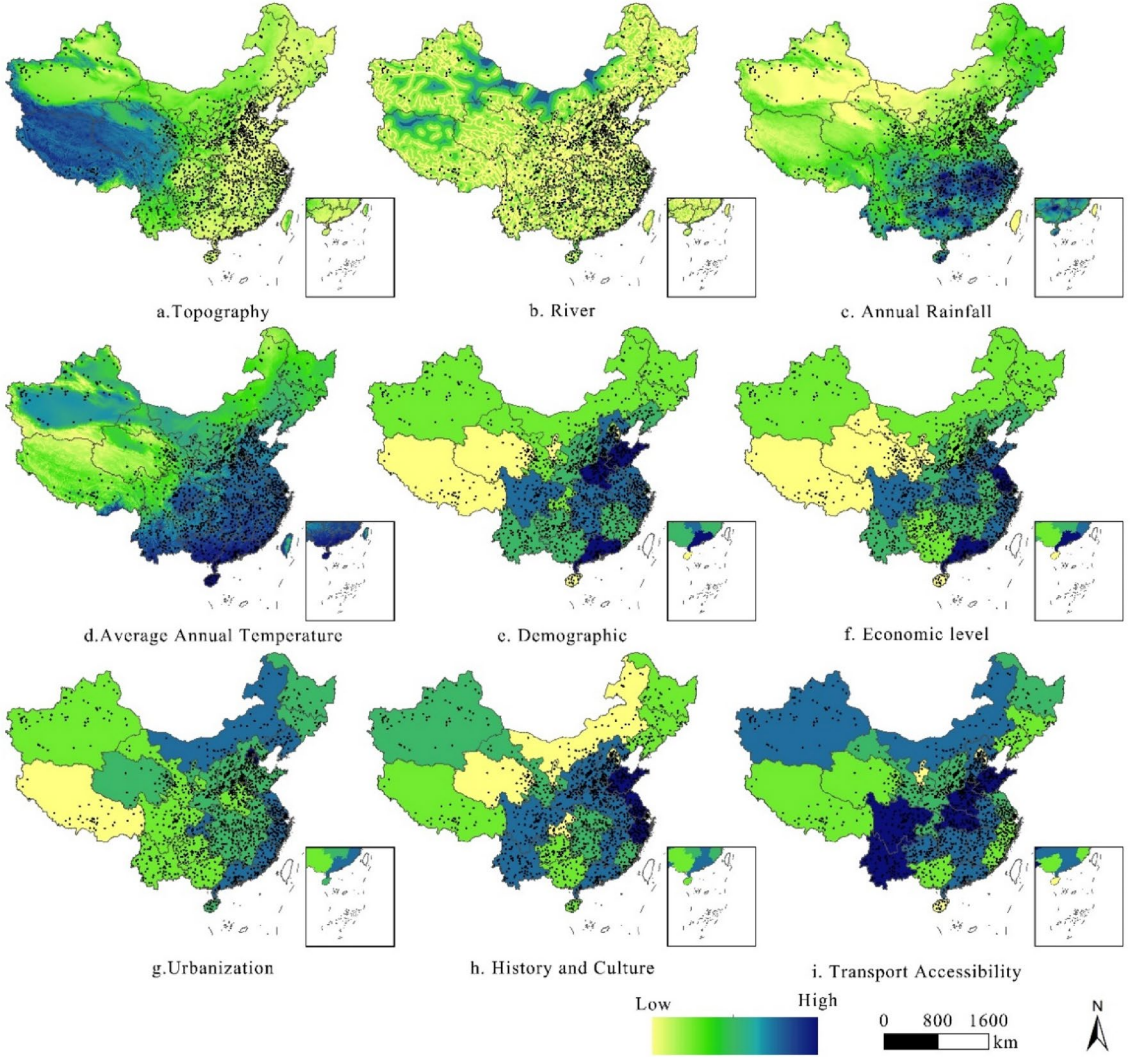
Overlay of Intangible Cultural Heritage Resources and Influencing Factors in China.

In this study, the influencing factors were analyzed in detail according to the order of the explanatory power of the influencing factors and in combination with the characteristics of the spatial distribution of intangible cultural heritage resources in China (Fig. 6).

The level of economic development has a significant impact on the spatial distribution of intangible cultural heritage resources, ranking first among all indicators. The protection and development of intangible cultural heritage resources depend on the strong driving force of the regional economy, and regions with good economic development have a relatively greater degree of cultural importance. Economically developed regions such as East China, South China and North China are able to provide more economic support for the protection of intangible cultural heritage resources than other parts of the country, and residents with higher economic resources have greater demand for cultural industries than more restricted residents. This heightened demand makes it easier to form local cultural industries related to intangible cultural heritage resources. In contrast, material support for intangible cultural heritage is comparatively weak in economically disadvantaged regions^39–41^.

Intangible cultural heritage resources are themselves an important expression of history and culture and have the second highest impact on all the indicators. Regions with a rich history and culture place greater value on intangible cultural heritage^41–43^. A rich cultural heritage not only provides the historical conditions for the preservation of a large number of intangible cultural heritage resources in the region but also facilitates the clustering of intangible cultural heritage resources in the region. Thus, this approach is more conducive to the formation of an atmosphere of resource protection and reduces the difficulty of resource protection.

Intangible cultural heritage is an embodiment of the hard work and wisdom of the Chinese people; these cultural resources are created by people, and the degree of population aggregation determines people’s ability to transform nature, both historically and in cultural development in modern times^42^. The more accessible a region is, the easier it is for intangible cultural heritage resources to be widely disseminated and to increase their cultural impact^44^, for example, in East, North and South China. In areas that are more isolated by transport options, there is less impact from foreign cultures, and it is easier for intangible cultural heritage resources, such as those in the Qiandongnan region, to maintain their uniqueness. Therefore, when analyzing the impact of transport access on intangible cultural heritage resources, it is important to consider not only areas with high accessibility but also intangible cultural heritage resource centers that are less exposed to external cultural influences. Upon further analysis, we found that high transport accessibility is more conducive to the formation and clustering of intangible cultural heritage resources.

Urbanization, on the one hand, can increase the cultural need for intangible cultural heritage resources in cities, thus attracting the attention of local residents and governments. On the other hand, rapid urbanization will accelerate the demise of traditional villages and, to a certain extent, diminish the original conservation conditions of some intangible cultural heritage resources, adding challenges to the transmission of intangible cultural heritage resources^43,44^. Upon further analysis, we found that urbanization is generally positively correlated with the preservation and development of intangible cultural heritage resources.

Areas along rivers with favorable climates and flat topographies are more suitable for human habitation^40^. The high-density zones in China are mostly located in the lower reaches of rivers with gentle terrain, so the formation of intangible cultural heritage resources is inextricably linked to natural geographic factors. When weighting the factors affecting natural conditions, climate > rivers > topography and geomorphology.

Climate has a significant impact on human production and life and is also an important natural factor in the location of human villages^44^. Agricultural conditions are better where there is more precipitation, and intangible cultural heritage related to agricultural cultivation techniques, such as the 24 seasons of the lunar calendar, which address the timing of agricultural cultivation, can develop in areas with high precipitation. Areas with low precipitation are often zones where animal husbandry is developed, and intangible cultural heritage unique to these zones, such as Mongolian animal husbandry techniques, can be formed. Temperature affects population concentration, and China’s population is concentrated in climatically favorable zones, such as eastern and northern regions. The hotter northwestern region not only has a smaller population distribution but also has sparse intangible cultural heritage resources. Further research has shown that temperature also plays an integral role in the formation of intangible cultural heritage resources. For example, the Northeast region is influenced by its cold climate, which has led to the development of traditional techniques for tanning animal hides and skins^43^.

Early humans lived mostly around rivers, which are important natural resources for production and life, and rivers therefore played an indispensable role in the formation and development of intangible cultural heritage^42–44^. A large number of intangible cultural heritages are related to rivers, e.g., dragon boat races and the Dai Water Festival. Topography and geomorphology also greatly influenced the production and life of the inhabitants historically, thus having a profound impact on the location of residential villages and cultural exchanges^41^. Local residents in open terrain easily communicate with one another, which is conducive to cultural development, while residents in closed terrain have less communication^42,43^, and local culture is less affected by foreign culture, which makes it easy to maintain the uniqueness of intangible cultural heritage resources.

### Interactive analysis

**Figure 7:**
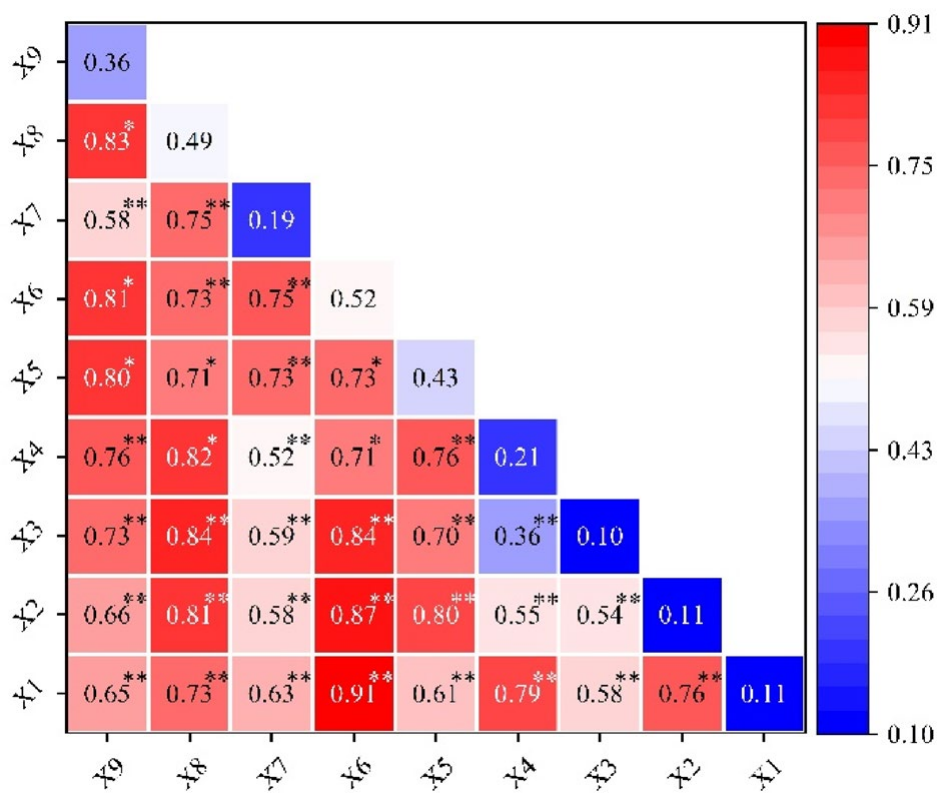
Interactive detection results of the influencing factors of China’s intangible cultural heritage resources.

The spatial distribution of China’s intangible cultural heritage resources is the result of the joint action of multiple factors, and their spatial distribution should be comprehensively explored from multiple perspectives (Fig. 7).

The interaction effect between topography and geomorphology and between the level of economic development was the most significant (0.91), with areas with flat topography and a greater level of economic development better able to support the preservation and development of intangible cultural heritage resources. The interactions between rivers and the level of economic development (0.87), climate and the level of economic development (0.84), and access to transport and the level of economic development (0.81) are also more prominent. As a result, the interaction coefficient of the level of economic development with other factors is much greater than the value of a single influencing factor. Riverine areas with high accessibility and humid climates have a high distribution of intangible cultural heritage resources.

The interactions between river and historical culture (0.87), climate and historical culture (0.84), transport accessibility and historical culture (0.83), and population and transport accessibility (0.80) are also more pronounced, and the interactions between natural and social factors are greater than the influence of a single factor, which suggests that they have a synergistic effect or a mediating effect on influencing intangible cultural heritage resources or mediating effects^40^. Areas with favorable climates, dense populations, developed economies and rich cultural heritage sites are more favorable for the clustering of intangible cultural heritage resources.

## Discussion

The spatial distribution of intangible cultural heritage resources in China varies significantly. This paper focuses on the spatial distribution of intangible cultural heritage resources as a whole and the ten major types of intangible cultural heritage in China at different scales. At the scale of the seven geographical subregions, East and North China are not only a cluster of intangible cultural heritage resources in general but also a cluster for all identified major types. This finding is consistent with the overall characteristics demonstrated by Li et al.^41^, who studied the spatial distribution of intangible cultural heritage resources in the Yangtze River Basin. However, at the provincial scale, the spatial distribution of the ten types of resources appears to be different from that of the combined resources overall. This finding suggests that the dominant factors influencing the formation and development of different categories of intangible cultural heritage resources differ.

In general, the dominant factor in the spatial distribution of intangible cultural heritage resources at larger scales, such as North and East China and the Yellow River Basin, is usually economic. This finding is basically consistent with the conclusions drawn by Pang et al.^40^, who studied the influencing factors of the spatial distribution of intangible cultural heritage resources in the Beijing-Tianjin-Hebei region, and Tian et al.^29^, who studied the influencing factors of the spatial distribution of intangible cultural heritage resources in the Yellow River Basin. In the past, previous researchers have analyzed only the factors influencing the spatial distribution of intangible cultural heritage resources in the entire region under study. On the one hand, it is easy to ignore the dominant factors of resource clustering in small-scale regions, such as the intangible cultural heritage resource clustering in Tibet, with Lhasa as the core, which is predominantly formed by ethnicity, and the intangible cultural heritage resource cluster in Sichuan, which is formed by topography. Analyses of the factors influencing the distribution of these small-scale regional resources are also necessary compared to those of intangible cultural heritage resources within the larger area of China. On the other hand, there is a lack of description of the spatial characteristics of different types of intangible cultural heritage resources; for example, traditional music, traditional dance and folklore in the Qiandongnan region are very densely distributed, while other types of resources are sparser. These findings could encourage the development of local cultural industries related to music, dance and folklore. Therefore, a discussion of the characteristics of the spatial distribution of different types of intangible cultural heritage resources is essential. In summary, when studying the spatial distribution of intangible cultural heritage resources, it is necessary to carry out detailed analyses at different geographical scales and for different types of resources to make the study more comprehensive and reasonable. By comparing the distribution of intangible cultural heritage resources in different regions and of different types, evidence-based proposals can be proposed to protect resources and promote the development of the cultural industry in light of the actual situation of unbalanced regional development.

By analyzing the factors influencing the spatial characteristics of intangible cultural heritage resources, the main reference is Wang et al.’s discussion of geodetectors. The method of detecting multiscale multifactor interactions differs from that used in previous single-factor studies^42^. Studies have shown that the synergistic effect of multiple factors, such as natural, economic and cultural factors, has a much greater impact on the spatial distribution of intangible cultural heritage resources than does a single factor^43^. The factors that dominate intangible cultural heritage resources vary across scales and types of intangible cultural heritage, but in general, social factors are more influential than natural factors. This study addresses the dominant factors of intangible cultural heritage resources at different scales in a region while also focusing on the influence of other factors on intangible cultural heritage resources. By analyzing the extent to which specific factors, both natural and socioeconomic, influence China’s intangible cultural heritage resources, it was concluded that the three influencing factors of economy, culture and transportation are more heavily weighted. However, scaling down to the seven geographic divisions and the provincial scale, we find that the weight of other influences changes significantly within a given region. By analyzing the distribution of intangible cultural heritage resources in regions such as Tibet and Sichuan, it is concluded that the weight of other influencing factors, such as topography, increases in specific small regions. We provide a rational explanation for this phenomenon. We propose a geographical basis for future research into the development of intangible cultural heritage resources and regional variability in different regions. Therefore, to protect cultural resources, it is necessary to clearly adhere to development strategies from a national perspective. Attention should be given not only to the impact of economic, cultural and transport accessibility on intangible cultural heritage resources but also to the role played by factors such as climate and topography.

This study mainly explores the overall spatial distribution of intangible cultural heritage resources in China and its influencing factors, which provides a reference for the exchange of intangible cultural heritage resources among regions and has positive significance for the protection, inheritance and development of intangible cultural heritage resources. In future research, indicators such as the period of formation of China’s intangible cultural heritage resources, the strength of government protection, and the participation of residents should be considered, as well as an in-depth exploration of the factors influencing each type of intangible cultural heritage resource. However, in general, this paper combines theory and practice to analyse the spatial characteristics of China’s intangible cultural heritage resources and study their influencing factors; moreover, this kind of targeted analysis of different agglomerations and integrated awareness of national planning have been less emphasized in previous research on intangible cultural heritage resources. The results of the study are also compared with previous discussions to enrich the theoretical results in this field, and the conclusions are scientifically sound and reliable.

## Conclusions and recommendations

### Conclusion

The analysis of the spatial distribution of China’s 3610 intangible cultural heritage resources and the factors influencing them led to the following conclusions:

1. In terms of spatial distribution, the spatial distribution of China’s intangible cultural heritage resources is uneven, with an overall agglomerative distribution, and the distribution of intangible cultural heritage resources in the north‒south direction is more prominent and significant than other axes. Overall, resources are predominantly distributed in the east and less dense in the west. There are more resources in the southern region than in the northern region.
2. In terms of the spatial distribution of intangible cultural heritage traditions overall and when broken down into their primary types, North and East China have always been areas with high density. The spatial distribution of intangible cultural heritage resources in China has generally formed two areas of high density and two areas of secondary density. There are also kernel density zones formed by Lhasa, the capital of the Tibet Autonomous Region, and Chengdu, the capital of Sichuan Province, which drive the neighboring areas.
3. In terms of spatial trends, there is a clear correlation between the distribution of intangible cultural heritage resources in China and the state of economic development and historical and cultural heritage; i.e., the more economically developed and culturally rich a region is, the more resources for intangible cultural heritage there are. At the provincial level, there are also unique clusters of intangible cultural heritage resources; i.e., due to the enclosed topography, areas such as Sichuan and Qiandongnan have been able to effectively reduce the impact of foreign cultures and maintain their local characteristics. Due to the large distribution of ethnic minorities, regions such as Qinghai and Tibet have formed clusters of intangible cultural heritage resources with ethnic characteristics.
4. The causes of the distribution characteristics of China’s intangible cultural heritage resources are complex and cannot be analyzed from a single factor alone. Among the influencing factors, social factors have a much greater impact on the spatial distribution of resources in China than natural factors. The dominant factors in the formation and development of different categories of intangible cultural heritage resources differ. The influence of multidimensional interactions on the spatial distribution characteristics of intangible cultural heritage resources in China is more obvious.

### Recommendations

According to the study of the spatial distribution of intangible cultural heritage resources and their influencing factors in China, the spatial distributions of different types and scales are somewhat different. We therefore make targeted recommendations to advance the preservation and development of intangible cultural heritage resources.

At the provincial scale, for regions with strong ethnic cultures or more closed territories, such as Tibet, Qiandongnan and Sichuan, thematic cultural activities should be created with the help of the local characteristics of different regions^46–48^. Awareness of intangible cultural heritage resources should be raised and more support provided to intangible cultural heritage bearers and intangible cultural heritage communities.

In the seven geographic subregions, for economically developed regions rich with intangible cultural heritage resources, such as North and East China, the coordination and cooperation between economic zones and intangible cultural heritage intensive zones should be strengthened to create a circle of intangible cultural heritage resources with regional characteristics. This development will promote the integration of culture and tourism^44,45^ and enhance awareness of the protection of intangible cultural heritage. Moreover, in the development process, attention should be given to avoiding the negative effects of excessive commercialization on the originality of intangible cultural heritage resources;

For intangible cultural heritage resources that are scattered and sparsely distributed at the national scale, financial investment in intangible cultural heritage resources should increase, internet technology should be used to increase publicity^49,50^, local intangible cultural heritage innovation and inheritance should be promoted, and creative products of intangible cultural heritage should be developed.

For more homogeneous resource agglomeration areas, such as Yunnan Province, traditional dance-based heritage resources should be vigorously developed to form a cultural industry base on characteristic traditional dance. The traditional dance of Yunnan Province will be vigorously publicized to increase the popularity of the city. Traditional dance and the local tourism industry should be closely integrated to attract national tourists and help high-quality local tourism development. For example, in Qinghai Province, full use should be made of the unique local resources of traditional music and operatic arts to create a base for classical music in Northwest China and develop a unique cultural industry. It is also possible to combine popular music with the unique ethnic styles of the Northwest to provide new life to local traditional music.

### Supplementary Information

Supplementary Information.

## Data Availability

All data generated or analysed during this study are included in this published article [and its supplementary information files].
