# Supplementary material for: Spatial distribution of intangible cultural heritage resources in China and its influencing factors
Source: Sci Rep. 2024 Feb 29;14:4960. doi: 10.1038/s41598-024-55454-2 (PMC10902377; doi:10.1038/s41598-024-55454-2)
Supplement: Supplementary file 1 — Supplementary Information. [file 41598_2024_55454_MOESM1_ESM.zip › Thesis-related datas/Supplementary figure S1~S6/Notes on the use of data.pdf]

Notes on the use of data

Figure 1 in the text shows the research framework diagram of the thesis, which was drawn through the software.

Using the spatial analysis tool ArcGIS10.8, we imported the relevant geographical coordinates of the non-heritage resources, and borrowed the relevant tools for spatial analysis to draw Figure 2~Figure 6.

Detailed production instructions for Figure 2 – 6:

The first step is to obtain the required China map maps through the China Standard Map Service website(http://bzdt.ch.mnr.gov.cn/). Review number: GS (2020) 4631, with no modifications to the map boundaries (this base map is used for figures 2, 4, 5 and 6)

The second step is to make the corresponding Figure 2 through the data import and coordinate entry functions produced by ArcGIS 10.8.

Step 3, through the kernel density analysis tool of ArcGIS10.8 to make Figure 4-Figure 6.
