# Supplementary material for: Spatial distribution of intangible cultural heritage resources in China and its influencing factors
Source: Sci Rep. 2024 Feb 29;14:4960. doi: 10.1038/s41598-024-55454-2 (PMC10902377; doi:10.1038/s41598-024-55454-2)
Supplement: Supplementary file 1 — Supplementary Information. [file 41598_2024_55454_MOESM1_ESM.zip › Thesis-related datas/Supplementary figure S1~S6/Supplementary figure S1í¬figure S6.docx]

Figure 1 - Figure 6


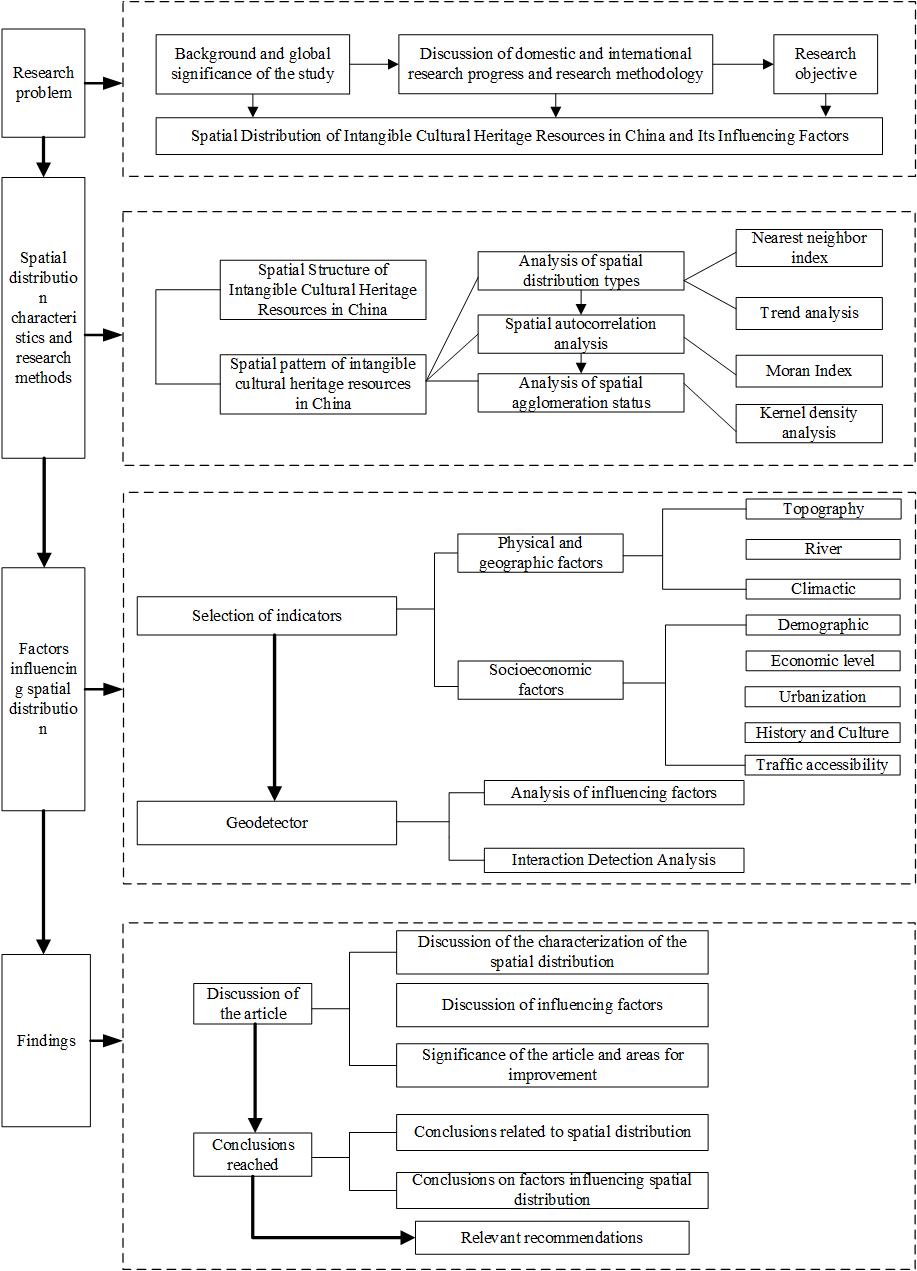


**Figure 1.** Research framework diagram.


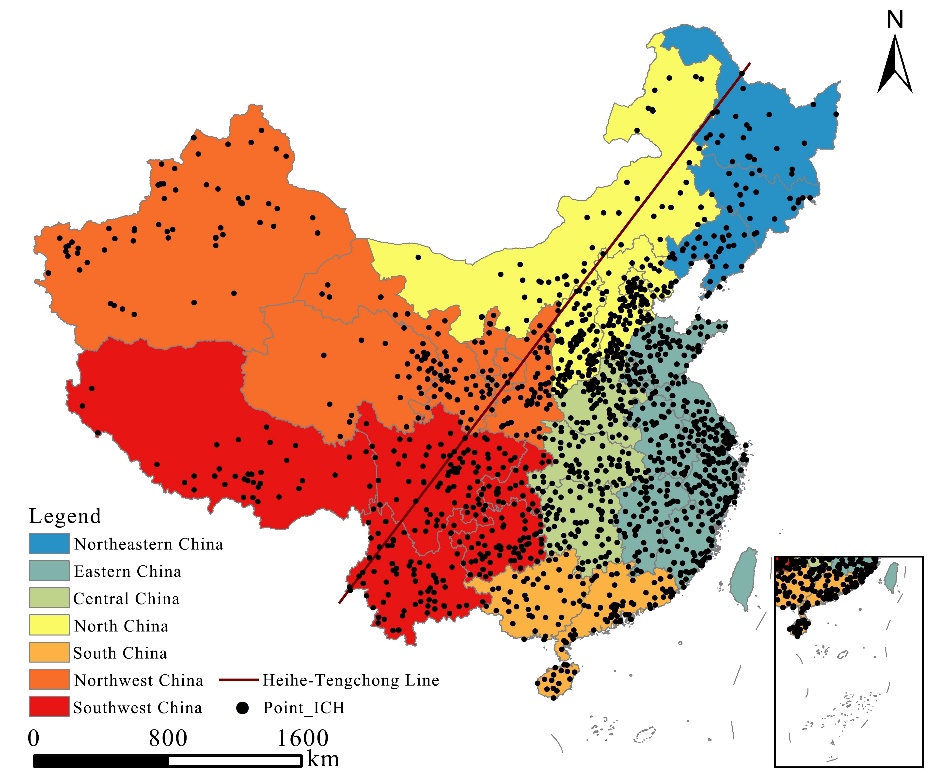


**Figure 2.** Spatial distribution of intangible cultural heritage resources in China.


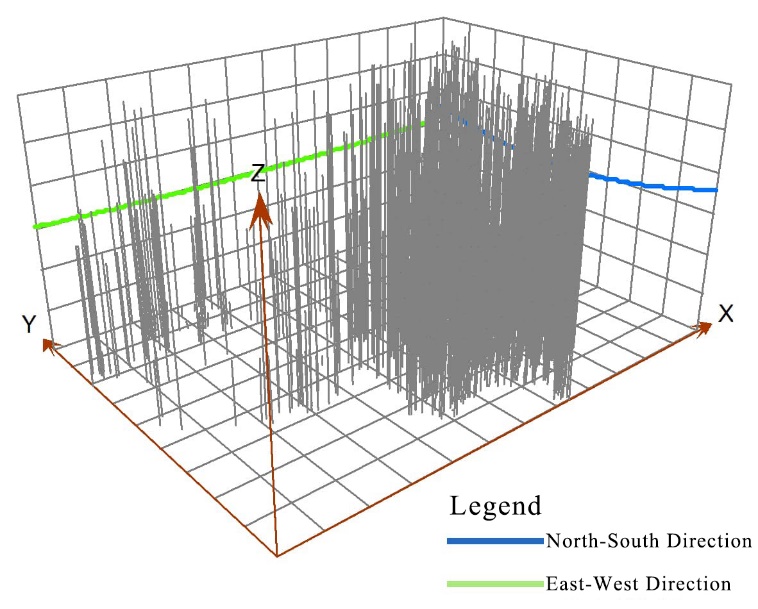


**Figure 3.** Surface fitting of general spatial trends in intangible cultural heritage resources.


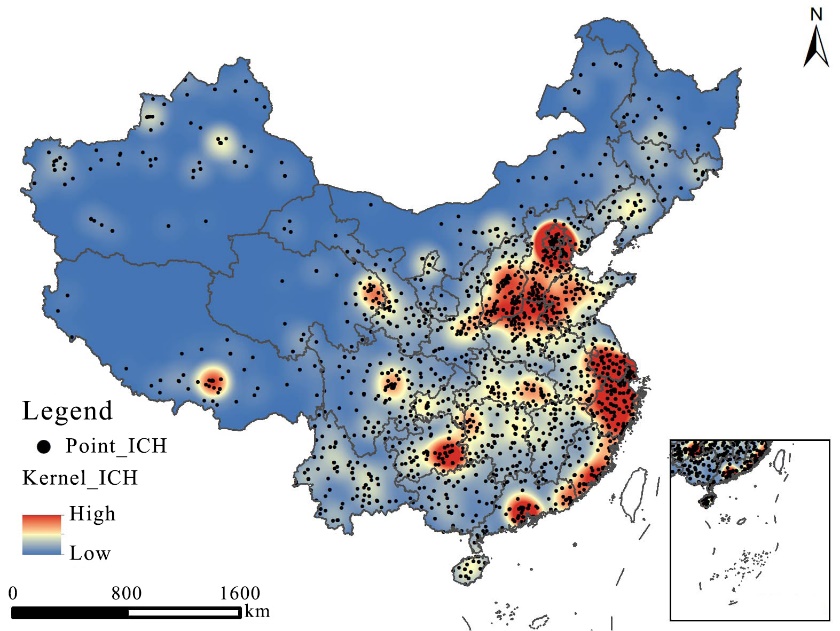


**Figure 4.** Overall distribution of kernel density of China’s intangible cultural heritage resources.


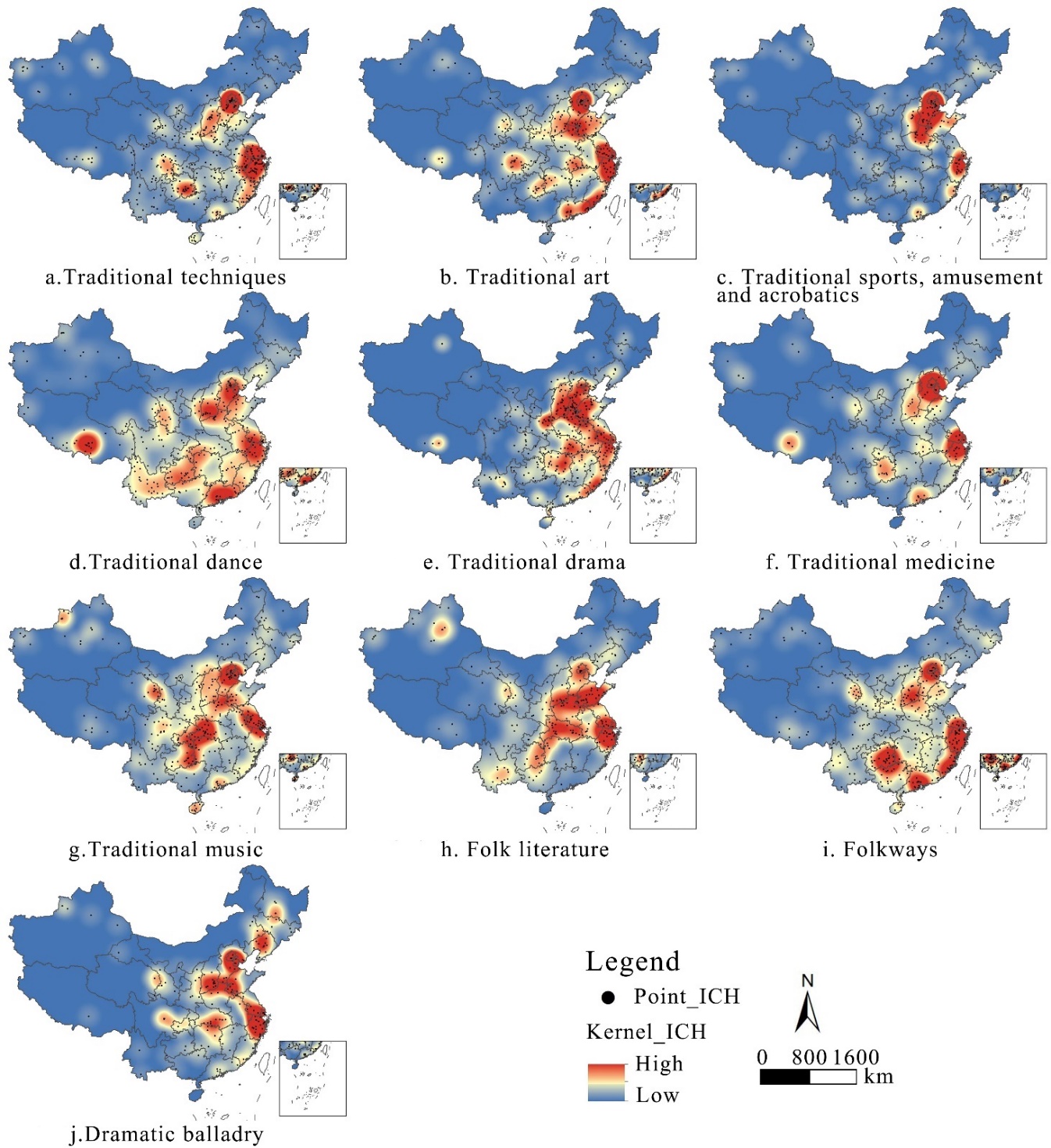


**Figure 5.** Distribution of kernel density for each type of China’s intangible cultural heritage resources.


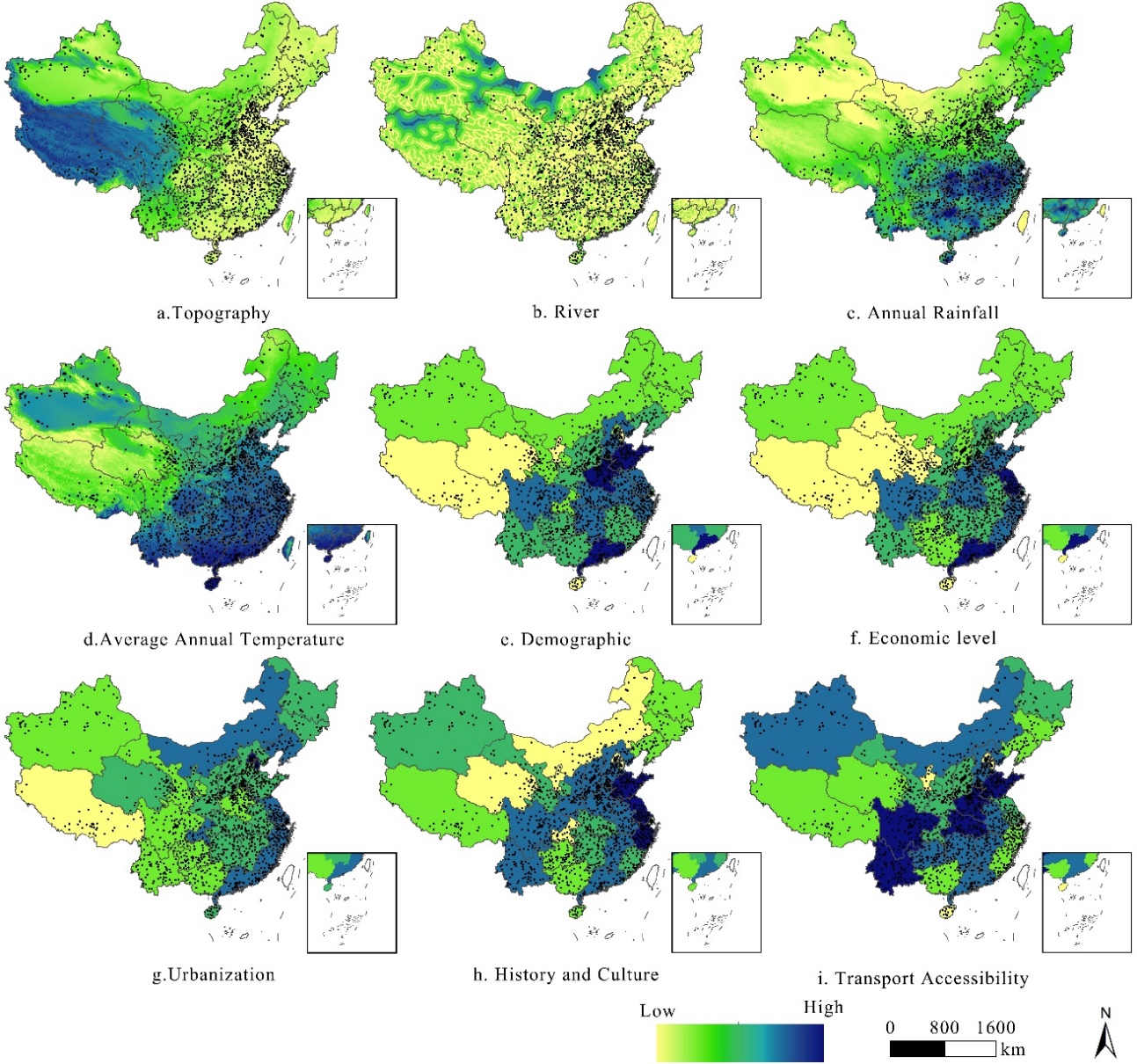


**Figure 6.** Overlay of Intangible Cultural Heritage Resources and Influencing Factors in China.
