# Supplementary material for: Spatial distribution of intangible cultural heritage resources in China and its influencing factors
Source: Sci Rep. 2024 Feb 29;14:4960. doi: 10.1038/s41598-024-55454-2 (PMC10902377; doi:10.1038/s41598-024-55454-2)
Supplement: Supplementary file 1 — Supplementary Information. [file 41598_2024_55454_MOESM1_ESM.zip › Thesis-related datas/Supplementary figure S7 and Supplementary table S4/Notes on the use of data.pdf]

Notes on the use of data

Using the collected data related to the influencing factors, we borrowed the Geodetector software to plot Table 4 and based on the results of the interactions between the influencing factors, we plotted Figure 7.
