# Supplementary material for: Spatial distribution of intangible cultural heritage resources in China and its influencing factors
Source: Sci Rep. 2024 Feb 29;14:4960. doi: 10.1038/s41598-024-55454-2 (PMC10902377; doi:10.1038/s41598-024-55454-2)
Supplement: Supplementary file 1 — Supplementary Information. [file 41598_2024_55454_MOESM1_ESM.zip › Thesis-related datas/Supplementary figure S7 and Supplementary table S4/Supplementary figure S7 and Supplementary table S4.docx]

**Table 4.** Explanatory power of influencing factors of China’s intangible cultural heritage resources.

| **Dimension** | **Physical and Geographic Factors** | | | | **Socioeconomic Factors** | | | | | |
| --- | --- | --- | --- | --- | --- | --- | --- | --- | --- | --- |
| **Targets** | X1 | X2 | X3 | X4 | X5 | X6 | X7 | X8 | X9 |  |
| **Q Value** | 0.108** | 0.109* | 0.103** | 0.206** | 0.431** | 0.519** | 0.192* | 0.486** | 0.363** |  |

Note: ** indicates significance at the 1% confidence level; * indicates significance at the 10% confidence level.


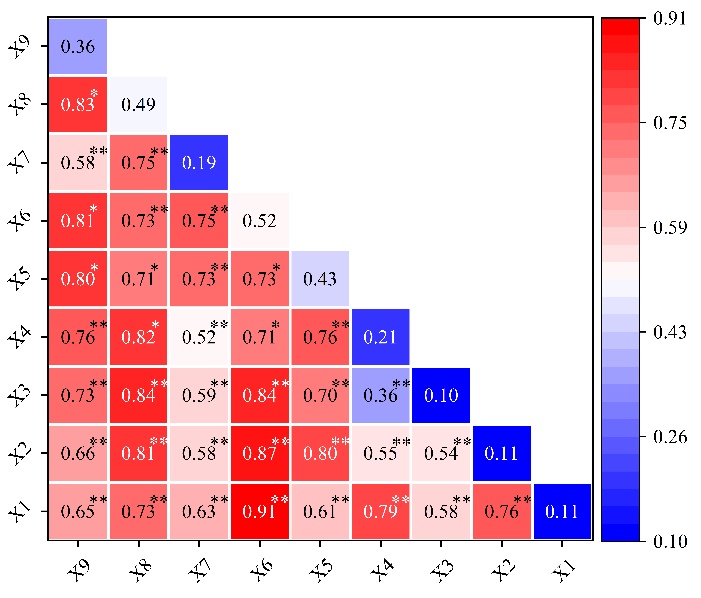


**Figure 7.** Interactive detection results of the influencing factors of China’s intangible cultural heritage resources.
