# Supplementary material for: Spatial distribution of intangible cultural heritage resources in China and its influencing factors
Source: Sci Rep. 2024 Feb 29;14:4960. doi: 10.1038/s41598-024-55454-2 (PMC10902377; doi:10.1038/s41598-024-55454-2)
Supplement: Supplementary file 1 — Supplementary Information. [file 41598_2024_55454_MOESM1_ESM.zip › Thesis-related datas/Supplementary table S1íótable S2 and table S3/Notes on the Data in TableS1í¬Table S3.pdf]

# Notes on the Data in Tables 1 、Tables 2 and Tables 3

Table 1 serves as a summary table, which is intended to be easy to read and to indicate the source of the information in the full text. Therefore the data in Table 1 has been described in detail in the paper.

The nearest-neighbour index operation in Table 2 is the same as the data and method used in Figures 1 to 5. The nearest-neighbour index in Table 2 is measured by the nearest-neighbour index function in the ArcGIS 10.8 software to measure the data used in Figures 1 to 5, and therefore the data in Table 2 is not duplicated for the provision of the data.

Regarding the source of data in Table 3, Table 3 has the same source of data as Fig. 1 ~ Fig. 5, and the same methodology has been used in Table 3 and Fig. 3, so please refer to the source of data in Fig. 1 ~ Fig. 5 for the data in Table 3.
