# Supplementary material for: Spatial distribution of intangible cultural heritage resources in China and its influencing factors
Source: Sci Rep. 2024 Feb 29;14:4960. doi: 10.1038/s41598-024-55454-2 (PMC10902377; doi:10.1038/s41598-024-55454-2)
Supplement: Supplementary file 1 — Supplementary Information. [file 41598_2024_55454_MOESM1_ESM.zip › Thesis-related datas/Supplementary table S1íótable S2 and table S3/supplementary table S1í¬table S3.docx]

**Table 1.** Indicator construction of influencing factors of China’s intangible cultural heritage resources

| **Dimension** | **Impact Factor** | **Evaluation Indicator** | **Data Source** |
| --- | --- | --- | --- |
| **Physical Geography** | Topography X_1_ | Average Elevation（m） | Geospatial data cloud platform access |
|  | River X_2_ | River Length（km） | China Basic Geographic Information System data |
|  | Climactic X_3_、X_4_ | Annual Rainfall ㎜ | China National Data Network |
|  |  | Average Annual Temperature ℃ |  |
| **Socioeconomic Factors** | Demographic X_5_ | Number of people in the area（ten thousand people） |  |
|  | Economic level X_6_ | Gross regional product（billions of yuan） |  |
|  | Urbanization X_7_ | Urban population as a proportion of total regional population |  |
|  | History and Culture X_8_ | Based on a combination of indicators such as number of local museums, number of art groups, etc. | Data from the National Statistical Yearbook of China |
|  | Traffic accessibility X_9_ | Road density |  |

**Table 2.** Summary of average nearest neighbors of intangible cultural heritage.

| **Typology** | **Techn-iques** | **Fine Art** | **Sports, Amusement and Acrobatics** | **Dance** | **Drama** | **Medicine** | **Music** | **Folk Literature** | **Folkways** | **Dramatic Balladry** | **Total** |
| --- | --- | --- | --- | --- | --- | --- | --- | --- | --- | --- | --- |
| **Quantity** | 629 | 417 | 166 | 356 | 473 | 182 | 431 | 251 | 492 | 213 | 3610 |
| **R-Value** | 0.35 | 0.44 | 0.53 | 0.56 | 0.47 | 0.41 | 0.51 | 0.56 | 0.47 | 0.49 | 0.24 |
| **Z Value** | -31.32 | -21.99 | -11.64 | -15.88 | -22.24 | -15.34 | -19.28 | -13.37 | -22.44 | -14.22 | -87.80 |
| **P Value** | 0.00 | 0.00 | 0.00 | 0.00 | 0.00 | 0.00 | 0.00 | 0.00 | 0.00 | 0.00 | 0.00 |

**Table 3.** Global spatial autocorrelation data table of China’s intangible cultural heritage resources.

| **Moran's I** | **Z Value** | **P Value** | **Global Distribution** | **Global Autocorrelation** |
| --- | --- | --- | --- | --- |
| 0.11 | 8.79 | 0.00 | clustering trends | significant spatial positive correlation |
